# 原发性肺癌术后继发或第二原发气管内肿瘤：系列病例报道及文献综述

**DOI:** 10.3779/j.issn.1009-3419.2023.101.20

**Published:** 2023-07-20

**Authors:** Wei HUANG, Peixin TAN, Zhongbiao XU

**Affiliations:** 510080 广州，南方医科大学附属广东省人民医院（广东省医学科学院）放疗科; Department of Radiation Oncology, Guangdong Provincial People’s Hospital (Guangdong Academy of Medical Sciences), Southern Medical University, Guangzhou 510080, China

**Keywords:** 肺肿瘤, 继发性气管内肿瘤, 第二原发性气管肿瘤, 气管转移, 气管癌, 放疗, 化疗, Lung neoplasms, Secondary endotracheal cancer, Second primary endotracheal cancer, Tracheal metastasis, Tracheal cancer, Radiotherapy, Chemotherapy

## Abstract

**背景与目的** 原发性肺癌根治术后出现继发或第二原发气管内肿瘤患者的临床特征、治疗手段及疗效、预后尚不清楚。本文将通过归纳总结相关病例，对以上问题进行详细阐述。**方法** 搜索广东省人民医院病历，筛选出5例肺癌术后出现气管肿物的患者，同时搜索PubMed从其他文献中筛选出9例病例。**结果** 本单位的5例患者中4例的气管内肿瘤被认为是继发于肺癌。在治疗方面，对这5例患者的气管内肿瘤分别进行了根治性切除术（n=2）、同步放化疗（concurrent chemoradiotherapy, CCRT）（n=1）、化疗（n=1）和姑息治疗（n=1）。接受CCRT治疗的患者获得最长的无进展生存期，为29.5个月。对于从其他文献中筛选到的9例患者，其中8例患者的气管内肿瘤被定义为转移。这9例患者的10个气管肿瘤分别进行了单独放疗（n=4）、CCRT（n=2）、单独化疗（n=2）和手术（n=2）。其中，1例单独接受放疗及1例接受CCRT的患者气管病灶均获得了完全缓解。**结论** 肺癌术后的患者出现气管内肿物应需引起足够重视。CCRT是原发性肺癌术后气管内新发肿瘤的有效治疗手段。

## Abstract (en)

**Background and objective** The clinical feature, treatment and outcomes of the patients with endotracheal cancer after radical surgery for primary lung cancer are unclear. This article will provide a detailed explanation of the above issues by summarizing the relevant cases. **Methods** We retrospectively reviewed five patients in Guangdong Provincial People’s Hospital and retrieved 9 cases from other literatures by searching PubMed. **Results** For five patients in out institute, 4 endotracheal cancers were considered as secondary to lung cancers. Radical stump resection (n=2), concurrent chemoradiotherapy (CCRT) (n=1), chemotherapy (n=1) and palliative care (n=1) were performed separately in 5 patients. The patient underwent CCRT achieved the longest progression-free survival of 29.5 months. For 9 patients retrieved from other studies, 8 endotracheal cancers were defined as metastases. Radiotherapy alone (n=4), CCRT (n=2), chemotherapy alone (n=2) or surgery (n=2) were performed separately in 10 lesions of 9 patients. 1 patient with radiotherapy alone and 1 patient with CCRT achieved complete response. **Conclusion** More attention should be paid to the abnormality of the trachea after surgery of lung cancer. CCRT may be a good choice for endotracheal cancers after primary lung cancer.

肺癌恶性度高、预后差。早期非小细胞肺癌（non-small cell lung cancer, NSCLC）在根治术后的复发转移率依然很高^[1]^。NSCLC引起的最常见的转移部位是骨骼、脑和肝脏，而气管的转移很少见。原发性肺癌导致气管内转移的发生率尚不明确，仅有一项研究报告^[2]^称，手术切除后NSCLC的气管转移总发生率为0.44%（6/1372）。因此，我们对原发性肺癌导致的气管内转移知之甚少。

第二原发性肺癌可以被定义为在不同位置和/或不同时间发展的两种独立的肺肿瘤^[3]^。因此，在第一原发肺癌根治性治疗后独立发生的气管内癌可以被定义为第二原发肿瘤，即使其组织学类型与原发肺癌相同^[4]^。但作出这一结论需非常谨慎并有充分的证据，否则会影响对肿瘤的分期及其后续治疗策略的判断。

最近，随着免疫治疗和靶向治疗与手术的联合应用，肺癌患者术后的生存期显著延长，气管内转移或继肺癌根治术后第二原发气管肿瘤越来越受到关注。然而，其治疗手段、疗效以及生存结局仍不明确。因此，我们报道了广东省人民医院原发性肺癌术后出现继发性或第二原发性气管内肿瘤患者的诊断和治疗方面的经验。此外，我们还回顾了相关文献，共同分析了本院及文献报道中患者的临床特征及相关治疗信息。

## 1 资料与方法

我们筛选了本院1991年1月至2019年12月诊断为原发性肺癌后出现气管内肿瘤的患者共12例。12例患者都在本院或院外接受了初始肺癌的手术治疗。其中，7例患者的诊断或治疗相关细节在病历系统中记录不清晰。因此，我们最终纳入了5例医疗记录和随访完整的病例进行分析。

所有原发性肺癌和气管内肿瘤的组织切片都经过一名具有20年工作经验的病理学主任医师仔细审阅并作出诊断。气管转移的诊断依据为气管病变与先前原发肺癌之间的病理组织学诊断相同。气管第二原发肿瘤的诊断依据为组织病理切片中发现气管原位癌或与原发肺癌不同的组织病理学诊断。放射科医生对入组患者的胸部计算机断层扫描（computed tomography, CT）进行复阅，以确认气管内肿瘤发生的日期和位置。我们以主动脉弓的上缘为界，将气管内肿瘤的位置分为气管上段或气管下段。我们将原发性肺癌和气管肿瘤发生之间的间隔定义为从原发性肺癌手术后到胸部CT上检测到气管内异常或气管肿瘤病理确认的时间间隔。我们对所有患者进行了随访并记录了生存情况，最后一次随访时间为2021年12月15日。

我们在PubMed中搜索气管内转移或原发性肺癌后第二原发气管肿瘤的相关文献，并检索了相关文章中的参考文献。从所有符合条件的文献中筛选出相关病例，并提取出患者的诊断、治疗和生存结局等详细信息。

## 2 结果

我院原发性肺癌术后出现气管内肿瘤的患者的临床特征分别见表1和表2。所有患者均为男性（n=5）。原发性肺癌诊断时的年龄为44-66岁（平均55岁）。5例患者中有4例有10-30包年的吸烟史（平均22.5包年）。患者的原发性肺癌的组织学类型均为鳞状细胞癌。原发性肺癌的位置分别位于左下叶（n=2）、右下叶（n=2）及右中叶（n=1），均无位于上叶。其中2例为中心型肺癌。肺癌的分期包括早期到中期均有分布（IB-IIIA期）。患者均接受了肺癌根治性手术，而术前均未行气管镜下肿瘤活检或经皮肺肿物穿刺活检。2例患者接受了术后化疗，1例患者接受术前新辅助和术后辅助放化疗。所有患者术后至少进行了1次胸部CT的复查，未见气管内肿瘤播散征象。

**表1:** 患者原发性肺癌的临床特征

Patient No.	Gender/Age (yr)^a^	Smoking history (Pack-year)	Histological type	Location	Ki67 of the tumor cell	TNM	Staging	Treatment
1	Male/48	30	SCC	LLL	6%	pT2aN0	IB	Lobectomy
2	Male/44	10	SCC	RLL (central)	5%	pT3N0	IIB	Lobectomy+CT^b^
3	Male/55	20	SCC	RML (central)	12%	pT3N2	IIIA	Lobectomy+CT+RT^c^
4	Male/66	30	SCC	RLL	5%	NA	NA	Lobectomy
5	Male/62	0	SCC	LLL	3%	pT2bN0	IIA	Lobectomy+CT^d^

^a^Age at the time of diagnosis of the primary lung cancer; ^b^Postoperative chemotherapy: Vinorelbine plus Cisplatin for 6 cycles; ^c^Neoadjuvant chemotherapy: Taxol plus Carboplatin for 2 cycles; Adjuvant chemotherapy: Taxol plus Carboplatin for 3 cycles; Postoperative radiotherapy: 40 Gy/20 F; ^d^Postoperative chemotherapy: Gemcitabine plus Carboplatin for 4 cycles. LLL: left lower lobe; RLL: right lower lobe; RML: right middle lobe; NA: not applicable; SCC: squamous cell carcinoma; CT: chemotherapy; RT: radiotherapy; CR: complete response; PR: partial response; TNM: tumor-node-metastasis.

**表2:** 患者气管内肿瘤的临床特征

Patient No.	ILT (mon)	Primary or secondary cancer	Histological type	Location	Local lymph node invasion	Synchronous metastasis	Treatment	Outcome
1	19.1	Secondary	SCC	Lower	4R, 4L	No	CCRT^a^	Metastasized to the right lung 29.5 mon after diagnosis of endotracheal cancer; alive 18 mon after surgery of right lung metastasis
2	56.3	Secondary	SCC	Upper	2L, 2R	Cervical lymph node	CT^b^	Died 5.8 mon after diagnosis of endotracheal cancer
3	18.1	Primary	SCC	Upper	NA	No	Surgery	Metastasized to the left lung 3 mon after diagnosis of endotracheal cancer; lost to follow-up after chemotherapy for metastasis
4	5.1	Secondary	SCC	Upper	NA	No	ST	Died 4.3 mon after diagnosis of endotracheal cancer
5	26.9	Secondary	SCC	Lower	NA	No	Surgery	Alive 10.8 mon after diagnosis of endotracheal cancer, but lost to follow-up afterwards

^a^Concurrent chemoradiotherapy: 60 Gy/30 F, Etoposide plus Cisplatin for 2 cycles; ^b^Vinorebine plus Carboplatin for 1 cycle. ILT: interval between occurrence of lung and tracheal cancer; mon: months; CCRT: concurrent chemoradiotherapy; NA: not applicable; LRFS: local recurrence-free survival; PFS: progression-free survival; OS: overall survival; ST: stent implantation.

肺癌和气管内肿瘤的发生间隔为5.1-56.3个月（平均25.1个月）。原发性肺癌的分期与间隔时间之间未见明显关系。在确认气管内肿瘤时，3例患者主诉咯血痰，2例患者无明显症状。患者的组织学类型均为鳞状细胞癌，其肿瘤增殖指数Ki67表达差异较大（3%-12%不等）。在5例患者中4例患者的气管肿瘤被定义为肺癌的气管转移瘤，而1例患者的气管肿瘤根据病理特征被定义为气管第二原发癌。图1显示了3例患者各自的肺癌和气管肿瘤的组织病理学特征。他们都表现出鳞状细胞癌的典型形态，而在第3例患者的气管肿瘤切片中观察到了原位癌的形态。因此，我们认为第3例患者的气管肿瘤是第二原发癌。

**图1:**
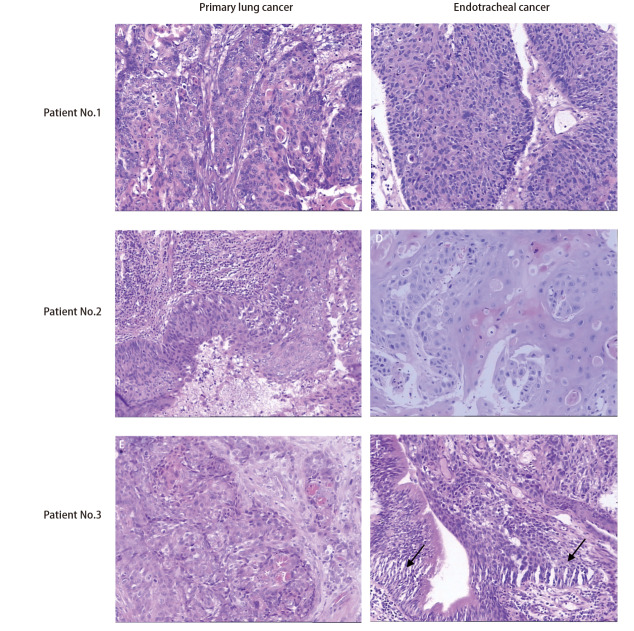
3例患者初治时肺癌及后续气管内肿物的病理学特征。病例1（A、B）和病例2（C、D）肺内及气管内肿瘤都表现出相似的典型的鳞状细胞癌特征。病例3（E、F）肺内及气管内肿瘤也表现出典型的鳞状细胞癌特征，但在气管内肿物的病理切片中发现了原位癌（箭头），提示气管内肿物为原发肿瘤（HE染色，×200）。

在5个气管肿瘤病灶中，3个病变位于气管上段，2个病变位于气管下段。2例患者的胸部CT扫描如图2所示。气管肿瘤有一些共同的特征：首先，它以结节形式生长；其次，在增强扫描下，气管肿瘤具有不同程度的强化；第三，气管肿瘤常容易侵犯气管软骨。5例患者中有2例同时显示纵隔淋巴结肿大。1例患者颈部淋巴结通过活检证实出现了转移。针对气管病灶，2例患者进行了根治性气管肿瘤切除术，其他3例患者分别接受同步放化疗（concurrent chemo-radiotherapy, CCRT）、单独化疗和支架植入姑息治疗。仅接受姑息性化疗或支架植入的患者在诊断为气管内转移后数月内死亡（分别为4.3和5.8个月）。1例患者术后3个月出现对侧肺转移，随后失访。另1例接受手术的患者在10.8个月后仍然活着，随后失访。接受CCRT的患者实现了29.5个月的无进展生存期，随后患者出现右肺转移，对右肺转移瘤进行手术后，患者继续存活了18个月。

**图2:**
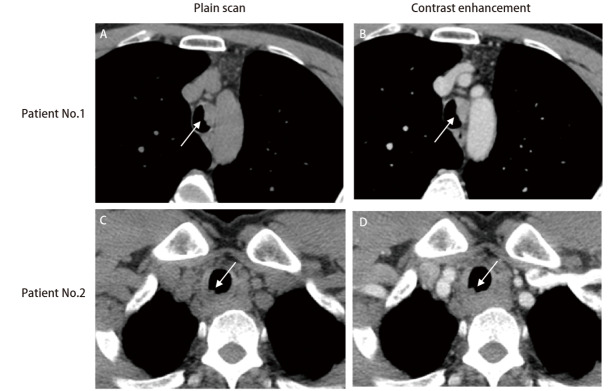
2例患者的胸部CT。A、B：1号患者的CT中发现左侧气管内存在一个贴壁生长的强化明显的结节（白色箭头），该结节侵犯了气管软骨导致软骨环中断；C、D：2号患者的CT中发现上段气管内的右侧壁存在一个轻度强化的小结（白色箭头）。

我们从PubMed网站上搜索并筛选出5项关于原发性肺癌术后出现气管肿瘤的研究。其中一项研究的语种为日语，被排除在外^[5]^。我们最终纳入了3项病例报告和1项系列报告，共包含了9例患者。患者特征如表3所示。1例患者为女性，8例为男性。9例患者的原发性肺癌中有2例为腺癌，其余为鳞状细胞癌。大多数患者为早期肺癌。针对原发肺癌，每例患者都进行了肺叶切除术（n=5）或全肺切除术（n=4）。肺癌和后续气管内肿瘤发生之间的平均间隔为21.4个月（范围：6-52个月）。只有1项研究报告了1例反复发生第二原发气管肿瘤的病例，而其他研究都将术后出现的气管肿瘤考虑为原发性肺癌引起的转移。有9个气管内肿瘤的病理组织学类型与其初始肺癌相同，而1个气管肿瘤的病理类型为小细胞癌，与原发的肺鳞状细胞癌不一致。5例气管肿瘤位于气管的上部，而其他则位于气管下部。对于气管肿瘤的治疗，患者分别进行了单独放疗（n=4）、CCRT（n=2）、单独化疗（n=2）及手术（n=2）。1例接受单纯放疗的患者和1例接受CCRT的患者获得了气管肿瘤的完全缓解。

**表3:** 文献报道中肺癌术后出现气管内肿瘤的患者特征

Author/year	Patient number/gender/age (yr)^a^	Primary lung cancer					Endotracheal cancer				
		Histological type	Position	Stage	Treatment		ILT (mon)	Histological type	Position	Treatment	Outcome^b^
Chong/2006^[2]^	1/Male/60	Adeno	LUL	IB	Lobectomy		26	Adeno	Upper	RT	33 mon, CR
	2/Male/68	SCC	LUL	IIA	Lobectomy		17	Squamous	Upper	RT	17 mon, PR
	3/Male/53	SCC	RLL	IA	Lobectomy		52	Squamous	Lower	Surgery^c^	68 mon, PD
	4/Male/66	SCC	RLL	IIIA	Lobectomy		8	Squamous	Lower	CT	18 mon, PR
	5/Male/53	SCC	RLL	IIIA	Pneumonectomy		11	Squamous	Upper	RT	20 mon, PD
	6/Male/64	SCC	LLL	IB	Pneumonectomy		41	Squamous	Upper	RT	52 mon, PR
Huo/2014^[22]^	7/Female/57	Adeno	RUL	IA	Lobectomy		7	Adeno	Upper	TC+CT	OS>24 mon
Youn/2013^[3]^	8/Male/51	SCC	RML	IIB	Pneumonectomy		6	Squamous	Lower	CCRT	NA
Lee/2015^[4]^	9/Male/62	SCC	LLL	IIA	Pneumonectomy+CT		18	Squamous	Lower	Surgery^c^+CT	CR
							28	Small cell	Lower	CCRT	CR

^a^Age at the time of diagnosis of the endotracheal cancer; ^b^The time by which each study was completed; ^c^Stump resection and bronchoplasty. Adeno: adenocarcinoma; LUL: left upper lobe; RUL: right upper lobe; TC: transbronchial cryotherapy; PD: progressive disease; OS: overall survival.

## 3 讨论

在这项研究中，我们报告了本院5例原发性肺癌术后继发性或第二原发气管肿瘤患者的诊断和治疗经验。此外，我们还回顾了相关文献以及其他研究中该类患者的临床特征。Chong等^[2]^报道的手术切除NSCLC后气管转移的总发生率为0.44%（6/1372）；鳞状细胞癌为0.77%，腺癌为0.18%。我院肺癌根治术后气管肿瘤（无论是转移还是第二原发）的发生率为0.76%（9/1178），略高于Chong等^[2]^的报道。我们试图想从患者原发肺癌的特点中寻找出发生气管转移或气管第二原发肿瘤的相关性，但是无论是肿瘤的生长位置、肺癌手术的切除方式以及肿瘤增殖指数Ki67与是否发生或是否更快发生气管原发或继发肿瘤似乎无明显关系。在分析了本院患者与其他单位患者的详细信息后，我们对原发性肺癌术后继发性或第二原发气管肿瘤总结了以下几个特征：首先，好发于中年患者（平均年龄58岁，范围44-68岁）；其次，大多数患者为男性（92.9%, 13/14）；第三，大多数患者有吸烟史（75%, 6/8），范围10-50包年；第四，大多数的原发性肺癌病理类型为鳞状细胞癌（85.7%, 12/14）。由此，我们推测有重度吸烟史和鳞状细胞肺癌的中年男性发生继发性或第二原发性气管癌的风险可能会增高。

在诊断气管肿瘤时，5例患者主诉咳血痰（36%, 5/14），1例患者主诉咯血（7%, 1/14），而大多数患者则无明显症状（57%, 8/14）。因此，我们建议患者无论是否有症状，都应定期复查胸部CT，并且应对气管的情况多加关注，避免气管肿物的漏诊。气管的微小病变或稍微增厚的管壁通常会被忽视或被视为管腔内的痰液（图3）。Chong等^[2]^建议建立影像学标准以区分气管内肿瘤或痰液，目前的判断方法包括：是否存在软骨环破坏、注射造影剂后观察气管病灶是否出现强化以及咳嗽后观察肿物位置是否有变化。此外，我们建议密切规律随访，如果强烈怀疑气管内肿瘤，则及时进行气管镜检查。

**图3:**
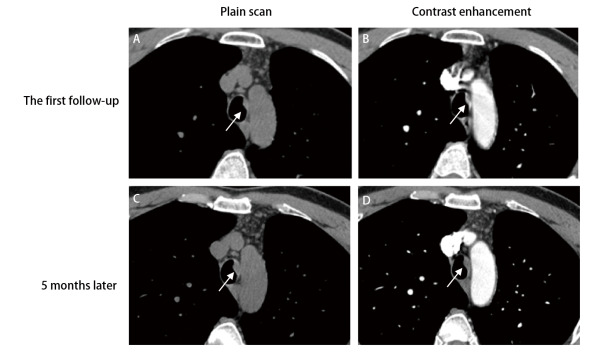
胸部CT展示气管内肿物的生长变化。A、B：在第一次的随访CT中，气管内的小结节表现为气管壁的增厚，被误认为是痰液（白色箭头；结节厚度约4.1 mm）；C、D：5个月后的随访CT可见气管内肿物明显增大（白色箭头；结节厚度约为5.3 mm），此时考虑该结节为肿瘤。

大多数第二原发肺癌与第一原发性肺癌之间有共同的遗传背景、相同的组织学类型^[6]^，因此二者难以区分。即使经验丰富的病理学医生，在没有显著的组织学或分子学差异的情况下，确定两种肺癌是独立的原发性还是转移性也存在高度的不确定性和分歧^[7]^。根据美国癌症联合委员会分期手册第8版，如果肿瘤起源于原位癌或具有不同类型的组织学、免疫学和分子亚型特征（例如，鳞状细胞癌的亚型）则可定义为独立的原发肿瘤^[8]^。由于原位癌是原发性肿瘤的金标准，而肿瘤的深度活检有助于发现原位癌，因此对气管肿瘤的诊断建议进行深度活检。由于不同的诊断会导致不同的治疗方案，对气管内肿瘤做出准确的诊断尤为重要。

目前共识认为两种具有不同驱动基因突变的肿瘤可被视为两种原发性肿瘤^[9]^。一项病例报道在检测到同一患者两处肿瘤出现相同表皮生长因子受体（epidermal growth factor receptor, EGFR）基因突变位点状态后，把气管内肿瘤定义为原发性肺腺癌的转移病灶^[4]^。由于驱动基因突变在腺癌中比较常见，因此，检测驱动基因的状态有助于区分原发或继发肿瘤，然而，在鳞状细胞癌和小细胞肺癌中，驱动基因的突变概率极低，检测驱动基因的意义不大。

肺癌纵隔淋巴结的解剖及引流情况已比较明确，而对气管淋巴管的解剖结构知之甚少。Borik等^[10]^的研究表明，右侧气管旁淋巴结链（2R和4R）参与构成气管的区域淋巴结和肺内淋巴结引流。这意味着肺和气管共享区域淋巴结的一部分，通过该区域淋巴结，来自肺部的癌细胞可能很容易迁移到气管。我院的2例患者在发生气管转移时同时出现气管旁和颈部淋巴结转移，这表明原发性肺癌可能通过淋巴系统迁移到气管内。

根治性手术被认为是可切除原发性肺癌的标准治疗方法，因为它可以带来更好的存活率（5年生存率为39%-47%，10年生存率为18%-36%）^[11⇓⇓⇓-15]^。对于因肺外肿瘤导致气管内转移的患者，手术切除可能是预防气管内再发转移的好选择^[16]^。但气管手术有相应禁忌证：存在多发阳性淋巴结、超过50%的气管受累以及不可切除的纵隔器官的侵犯^[14]^。此外，为了确保肿瘤的完整切除，至少50%的气管可能会被切除，最终导致吻合口高张力和生活质量的降低。这也是大多数原发性鳞状细胞癌气管肿瘤患者不宜手术的重要原因^[2,17,18]^。本研究中4例患者接受了手术。然而，由于随访时间短、样本量小，我们无法得出任何关于手术疗效及术后生活质量评价的结论。但我们注意到，这些患者在气管手术后不久就出现了转移。因此，我们认为在对有肺癌病史的患者进行气管肿瘤根治性手术时应谨慎。

放疗常用于无法切除的气管肿瘤患者或作为肿瘤进行不完全切除后的辅助治疗。文献^[19]^报道放疗可提高所有气管肿瘤患者的生存率，尤其是组织学为鳞状细胞癌、肿瘤局部侵犯较广以及肿瘤未完整切除的患者。鳞状细胞气管肿瘤的放疗剂量一般为60-70 Gy（中位生存期为24个月；5年生存期为27%）^[20]^。本研究中的4例患者接受了气管肿瘤的单纯放疗，中位无进展生存期为30.5个月，截至末次随访时间均未出现死亡。

单独化疗不是气管肿瘤的根治性治疗手段，而CCRT联合治疗可能是治疗气管肿瘤达到完全缓解的有效方法^[21]^。本研究中2例患者接受了CCRT并取得了临床完全缓解。迄今为止，这2例患者已有47.5个月没有出现复发。我们认为，CCRT可能比单独放疗能获得更长的生存期。因此，CCRT可能是原发性肺癌后气管内肿瘤的有效治疗手段。

气管肿瘤的其他局部治疗方法，如近距离放射治疗、光动力疗法、支气管镜电烙术和激光消融术等也被报道是有效的治疗手段^[22⇓⇓⇓-26]^。然而，大多数被用作姑息治疗，需要进一步的研究来验证这些方法的有效性。

我们的研究有一定的局限性。首先，由于原发性肺癌手术后继发或第二原发气管肿瘤的发病率较低，导致本研究的样本量较小；其次，因为活检的组织样本太少或年代久远，难以增加检测项目，如基因检测等，以进一步鉴别继发性或原发性气管内肿瘤，明确诊断。

综上，本院出现肺癌根治术后气管肿瘤的患者和其他研究报道的患者在诊断、治疗和预后方面均表现出相似的特征。在肺癌治疗后的随访中，应更加注意气管异常情况的观察，尽早发现及明确气管肿瘤的诊断。CCRT可能是原发性肺癌根治术后出现气管肿瘤的有效治疗手段。针对这一类疾病，未来需要更多的研究。

**Competing interests**

The authors declare that they have no competing interests.
